# Detecting ear lesions in slaughtered pigs through open-source convolutional neural networks

**DOI:** 10.1186/s40813-025-00442-9

**Published:** 2025-05-23

**Authors:** Matteo D’Angelo, Domenico Sciota, Anastasia Romano, Alfonso Rosamilia, Chiara Guarnieri, Chiara Cecchini, Alberto Olivastri, Giuseppe Marruchella

**Affiliations:** 1 Department of Bioscience and Agro-Food and Environmental Technology, University of Teramo, Via R. Balzarini, 64100 Teramo, Italy; 2 Department of Veterinary Medicine, University of Teramo, Loc. Piano d’Accio, 64100 Teramo, Italy; 3 Istituto Zooprofilattico Sperimentale della Lombardia e dell’Emilia-Romagna “Bruno Ubertini”, 25124 Brescia, Italy; 4 Local Health Unit Authority, 41121 Modena, Italy; 5 Local Health Unit Authority, 63100 Ascoli Piceno, Italy

**Keywords:** Pig, Slaughterhouse, Animal welfare, Ear lesions, Artificial intelligence

## Abstract

**Background:**

Ear biting is a damaging behavior of pigs, likely triggered by a genetic predisposition, previous health issues and/or poor environmental conditions. The accurate assessment of animal health and welfare relies on the systematic gathering of data about animals, resources and management. In this respect, slaughterhouse surveys offer valuable insights, as distinct tail and skin lesions can act as ‘iceberg’ parameters, suitable to estimate welfare during the entire animals’ lifecycle. However, the routine recording of lesions is often costly and time-consuming, making it unfeasible in high-throughput abattoirs. This study aims to train open-source convolutional neural networks for detecting ear biting lesions in slaughtered pigs, as a pre-requisite for a systematic and cost-effective welfare monitoring.

**Results:**

A total of 3,140 pictures were employed to train and test open-source convolutional neural networks. Investigations were carried out by three veterinarians, who agreed to assess porcine ears using a simplified method, to minimize inter-observers’ variability and to facilitate the convolutional neural networks’ training: a) healthy auricles (label 0); deformed auricles displaying alterations in their contour due to real lesions (label 1); postmortem artefacts due to slaughtering (label 2). The entire dataset (training set and test set) was evaluated by one observer, while a supplementary set of 150 pictures was assessed by all veterinarians. Overall, the agreement among observers was very high (*Cohen*’s kappa coefficient > 0.88). Moreover, convolutional neural networks’ performances appeared suitable when compared with veterinarians: overall accuracy 0.89, specificity 0.96, sensitivity 0.86, agreement with each individual observer 0.79 (*Cohen*’s kappa coefficient).

**Conclusions:**

Open-source convolutional neural networks can achieve good performances, especially when the task is strictly defined and rather easy. Valuable experiences are being gathered about the routine application of artificial intelligence-powered tools in pig abattoirs. We consider that such tools will likely enable the systematic collection of data, addressing the distinct needs of stakeholders in a cost-effective manner.

## Background

Ear biting is a damaging behavior in pigs that typically emerges between 6 and 12 weeks of age, likely triggered by a genetic predisposition, previous health issues (e.g., exudative epidermitis or sarcoptic mange) and/or poor environmental conditions. Lesions resulting from ear biting usually begin at the auricle base or tip and can extend to affect the entire pinna in more severe cases. The prevalence of ear biting usually ranges from 10 to 25% at slaughter [1, 2]. These lesions often start as small scratches or bite marks, later becoming covered with a crust of inflammatory debris, and may ultimately lead to near-complete loss of the pinna [3]. The observation of ear biting lesions at the slaughterhouse can be a reliable indicator for assessing on-farm pig welfare [4–6].

Animal welfare is perceived as a relevant challenge within the European Union, prompting stringent regulations and ongoing improvements in livestock production [7]. Over time, the idea has emerged that both negative and positive experiences should be considered when evaluating whether an animal has “a life worth living” [8]. Aligned with these developments, the definition of animal welfare provided by the World Organization for Animal Health is among the most widely accepted: “*An animal experiences good welfare if the animal is healthy, comfortable, well nourished, safe, is not suffering from unpleasant states such as pain, fear and distress, and is able to express behaviours that are important for its physical and mental state*” [9]. Notably, animal welfare is understood as an intrinsic characteristic of the animal itself, subject to fluctuations and quantifiable through the combination of different measures [10]. In this context, animal-based indicators are recognized as the most appropriate and are included within widely adopted welfare assessment protocols [11, 12]. Several methods have been developed to score tail and skin lesions, which can act as ‘iceberg’ indicators of broader welfare issues on farms [13, 14]. Skin lesions occurring at different stages of pig farming remain still detectable at slaughter, making their assessment useful for estimating welfare outcomes across the animals’ entire lifecycle [15].

The systematic record of lesions at postmortem inspection is costly and time-consuming, making it unfeasible in high-speed slaughter chains. In addition, intra- and inter-operators’ variability raises some concerns about the consistency of data, event when simplified methods are employed (e.g., presence/absence of distinct pathological findings) [13, 16, 17]. The implementation of artificial intelligence (AI)-powered technologies could address the above issues, particularly in high-throughput abattoirs, where huge numbers of animals are processed daily [18–20]. Artificial intelligence is a discipline aiming to develop intelligent agents and it is massively impacting upon human activities. When considering medical sciences, the recent success of AI in pathology and diagnostic imaging largely results from the development of convolutional neural networks (CNNs), which can be effectively trained to solve image understanding challenges. Likewise, AI-based tools have been developed to detect lesions and carcass contamination in slaughtered animals [20–23].

With this in mind, this study aims to train CNNs for detecting ear biting lesions in slaughtered pigs, using open-source and freely available tools. The performances of such CNNs are compared to those of veterinarians, regarded as the gold standard. The systematic detection of ear biting lesions could contribute to assess pig welfare on farm.

## Materials and methods

### Animals and photo acquisition

A total of 1,570 porcine carcasses were randomly selected for this investigation, which was carried out between January 2023 and August 2024. The pigs, aged 9 to 11 months and weighing 150 to 180 kg, were slaughtered at three abattoirs located in Central and Northern Italy, under different field conditions (e.g., lighting, background, processing speed).

**Fig. 1:**
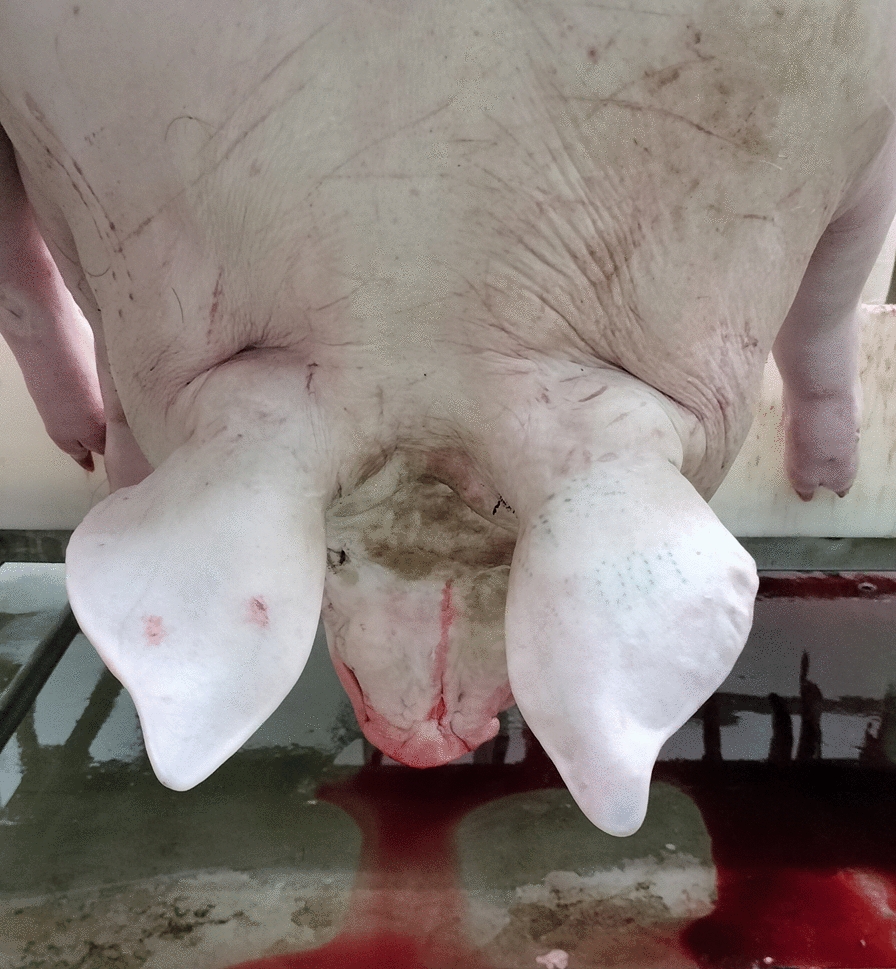
A model image used to develop the CNNs for automated ear lesion detection in this study. The photograph captures the head (the outer surface of both auricles is clearly visible), as well as the neck and the cranial portion of the trunk. In this case, the distal parts of the forelimbs are also visible. The image was taken along the slaughter chain, without using any panel as a background

Carcasses were photographed along the slaughter chain, after passing through the scalding tank, flaming and brushing. Pictures were taken by different operators using smartphone cameras (i-PhoneSE, i-Phone13, OPPO Reno8 Lite, Honor X8) and showed the head and the neck (see Fig. 1 for details).

### Picture labelling

Picture labelling was carried out by three veterinarians (namely, observer A, B and C), who agreed to classify each porcine auricle as follows:

•Healthy auricles showing a normal silhouette (label 0);
•Deformed auricles displaying alterations in their contour, due to real lesions and regardless of their severity (label 1);
•Auricles showing postmortem artefacts, such as cuts or strains after brushing (label 2).

**Fig. 2:**
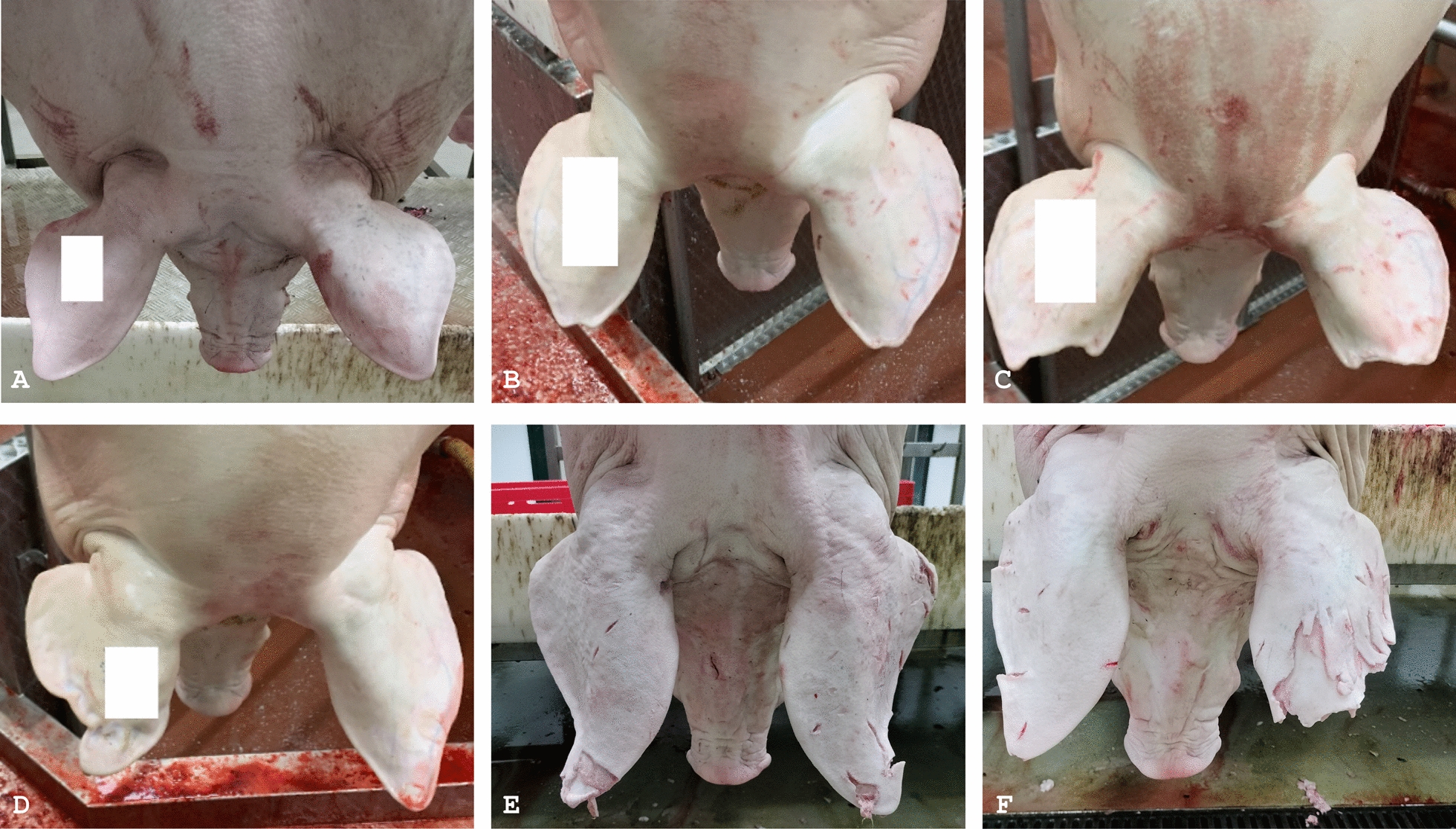
Representative examples of healthy, diseased and altered porcine ears. **A** both pinnae appear healthy and show a normal silhouette; **B** mild ear lesions, small tissue loss affecting the apex of both auricles; **C** severe tissue loss affecting both ears; **D** the right ear appears crumpled, like the outcome of an otohematoma; **E** and **F** alterations of the auricular profile, artefacts due to the slaughtering process, particularly after carcass brushing to remove bristles. In figures **A**–**D**, white boxes have been added to hide the tattoos, which bring the alphanumeric code indicating the country and herd of origin

Label 1 was assigned even when lesions and artefacts were both present in the same auricle. As a matter of fact, tissue losses affecting the ear profile were only considered in this investigation (explanatory pictures are provided in Fig. 2).

All observers (A, B and C) provisionally evaluated 300 pictures (i.e., 600 auricles) to estimate their mutual agreement.

One veterinarian (observer A) analyzed 1,420 pictures (i.e., 2,840 auricles), to train the CNNs and to test their performances.

A supplementary set of 150 pictures (i.e., 300 ears) was finally assessed by the CNNs and by all veterinarians (A, B and C), to further estimate their mutual agreement and the CNNs’ performances.

All images were sequentially numbered and labels recorded on a *Microsoft® Excel* spreadsheet, to train the CNNs and for statistical analysis.

### Deep learning-based method employed

#### Pre-processing of pictures

**Fig. 3:**
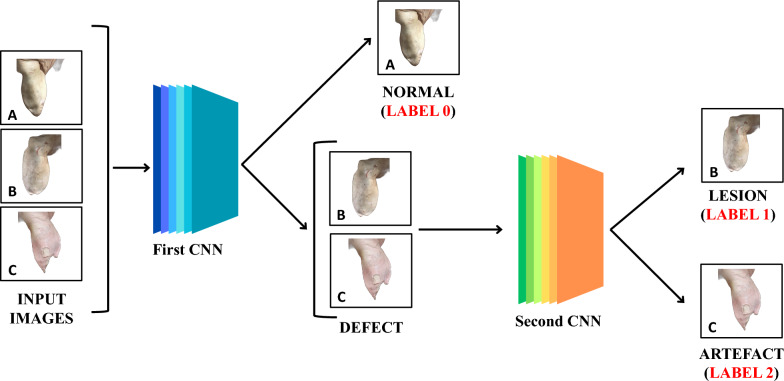
Overview of the entire AI-based process. Pre-processed images are analyzed by the first CNN (“*Sequential model*”, available at https://keras.io), which discriminate between “healthy” ears (**A**, label 0) and “defects” (**B**, **C**). The latter are further analyzed by the second CNN (“*Sequential model*”, available at https://keras.io), which discriminates lesion (**B**, label 1) from artefact (**C**, label 2)

Images were processed through ad hoc* Python* scripts and open-source tools (see Fig. 3 for details). First, the algorithm checked the type of the input pictures, the following formats being considered suitable: png, jpg and jpeg. Where needed, pictures of different format were converted in jpg files using *Python Imaging Library*. Thereafter, the *OpenCV library* (https://opencv.org) was used to center the main subject (i.e., the carcass) on the screen and to distinguish it from the background. To do this, *OpenCV* first converted the image to greyscale and then to RGB (i.e., red, green, blue). The background was removed using the "rembg" tool (available at www.github.com/danielgatis).

The *OpenCV* library standardized the images by taking the lowest and most lateral points of the carcass as edges, to ensure consistent formatting. Pictures were then subdivided into four parts; the lower ones contained one auricle each and were retained. At this point, a second round of background removal was performed with the “rembg” tool, to delete the area outside the ears. Finally, processed images were moved to a designated input folder for the dataset to be further analyzed.

#### Dataset

The entire dataset consisted of 3,140 pre-processed images, as each auricle was given individually to the CNN.

The training set consisted of 1990 images (63% of the dataset) and was employed during the training stage, provided to the CNNs along with labels assigned by the observer A. The test set consisted of 850 images (27% of the dataset) labelled by the observer A, and it was shown to the CNN during the inference stage, when no weight could be changed. Therefore, CNNs’ predictions were compared with the observer A’s labels.

In addition, the CNNs’ performances were assessed on a supplementary test set of 150 pictures (i.e., 300 ears), by comparing their predictions with all veterinarians’ labels (observers A, B and C).

#### Convolutional neural network

An open-source CNN (“*Sequential model*”, available at https://keras.io) was employed. In detail, such CNN consists of six alternate layers: (a) three convolutional layers, which extract features from the input image and give a feature map as outcome; (b) two pooling layers, serving to optimize and summarize the list of features generated by the convolutional layers; (c) the fully connected layer, which analyzes the entire list of attributes and gives a prediction (i.e., label assignment) as a result. When the patterns of interest are recognized, the image is labeled as “normal” (label 0) or “defect”. In the latter scenario, the image is further analyzed by a CNN (see Fig. 3 for details), identical to the previous one, but ad hoc trained to distinguish lesions (label 1) from artefacts (label 2). The CNNs’ predictions were recorded on a *Microsoft® Excel* spreadsheet and submitted to statistical analysis.

#### Statistical analyses

Inter-raters’ reliability was provisionally computed (*Cohen*’s kappa coefficient). Therefore, CNN’s performances were assessed in term of accuracy, specificity and sensitivity, when compared with the veterinarian (observer A), who evaluated the entire dataset. Finally, the agreement between CNNs, on one side, and each individual operator (observer A, B and C), on the other, was computed on a supplementary set of pictures (300 ears), in terms of accuracy, specificity, sensitivity, and agreement (*Cohen’*s kappa coefficient).

## Results

The agreement among the three different observers was provisionally computed on 600 randomly selected auricles, *Cohen*’s kappa coefficient ranged between 0.88 (observer C *vs* A; observer C *vs* B) and 0.92 (observer A *vs* B).

According to observer A’s assessments, the training set included 1,020 normal ears, while the auricles’ silhouette appeared altered in 970 pictures. In detail, 840 ears showed lesions, while artefacts due to slaughter-related procedures were seen in 130 cases. Considering the test set, the observer A classified 641 ears as normal (75.41%), and the remaining 209 ears (24.59%) as altered. In detail, lesions were detected in 90 cases (10.58%), while artefacts due to slaughter-related procedures were identified in 119 cases (14%). Overall, the CNNs achieved an accuracy of 0.88, a specificity of 0.89, and a sensitivity of 0.87 on the same test set (Table 1).

**Table 1:** CNNs’ performance on test sets

	Accuracy	Sensitivity	Specificity
Test set (850 ears)	0.88	0.87	0.89
Supplementary test set (300 ears)	0.89	0.86	0.96

The CNNs’ accuracy, sensitivity and specificity remained consistent both when compared to observer A—who contributed to their training (test set)—and when compared to all three observers involved in the study (supplementary test set)

**Fig. 4:**
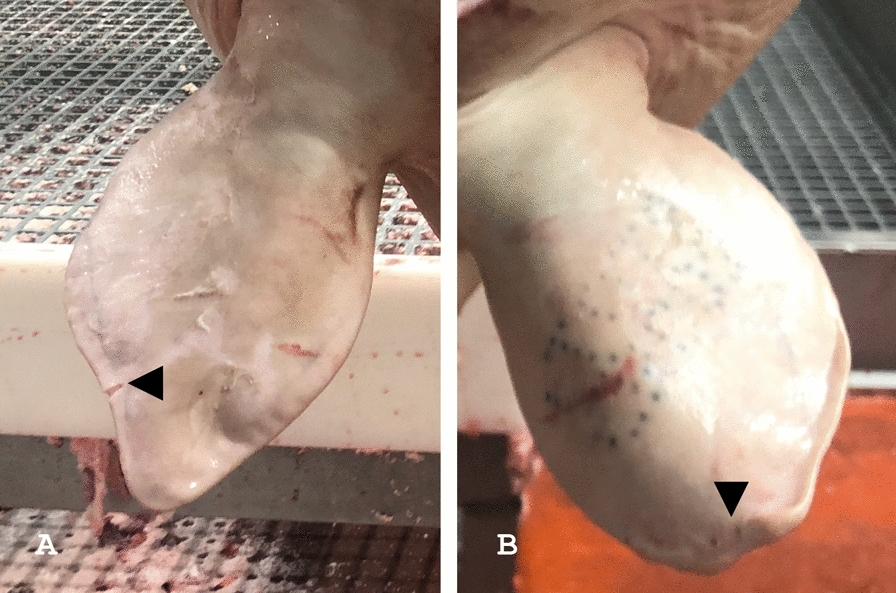
Representative examples of disagreement among observers. **A** Observer **B** disregarded a small artefact (black arrowhead), considering that it did not remarkably alter the ear profile. **B** Observer **C** identified a lesion at the ear tip (black arrowhead), while observers **A** and **C** deemed it was healthy and slightly rotated backward

The agreement among the observers was further assessed on the supplementary test set (i.e., 300 ears), *Cohen*’s kappa coefficient ranging between 0.93 (observer C *vs* A; observer C *vs* B) and 0.96 (observer A *vs* B). Observers agreed to identify 185 healthy ears (label 0), 85 real lesions (label 1) and 16 artefacts (label 2), while their rating was inconsistent in 14 cases. When focusing on these latter 14 ears, observer C disagreed with the other two in 8 cases, observer B in 4 cases, and observer A in 2 cases. In most instances (n = 8), discrepancies arose from the misclassification of healthy ears as pathological, or vice versa. In the remaining 6 pictures, healthy (n = 3) or pathological (n = 3) ears were misclassified as artefacts (see Fig. 4 for details).

**Fig. 5:**
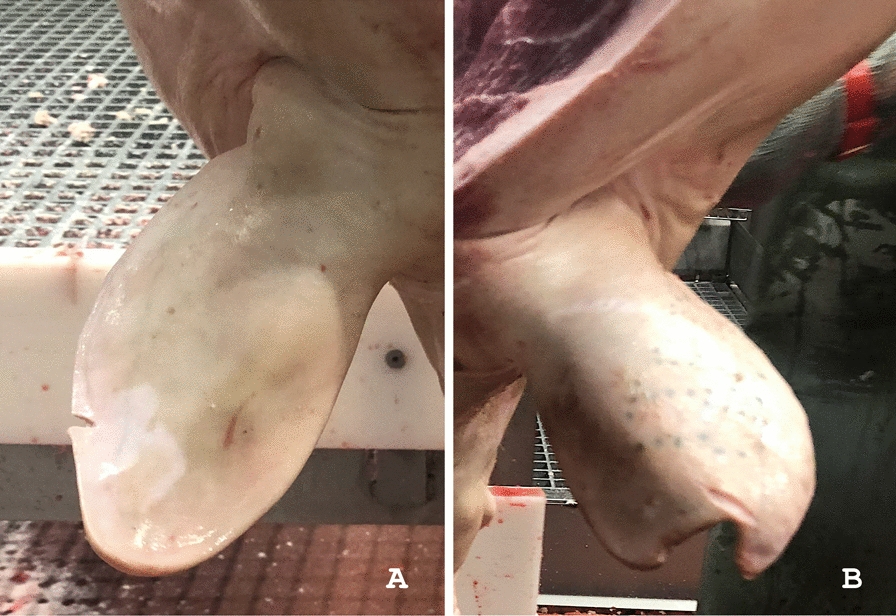
Representative examples of misclassification provided by the CNNs. This panel illustrates some CNNs’ mistakes: **A** the ear profile is affected by a small cut, which was consistently detected by the observers as an artefact (label 2), whereas it was ignored by the CNN (label 0); **B** all the observers agreed to classify this auricle as affected by a real lesion (label 1), whereas the CNN considered it as an artefact (label 2), likely because of the unusual appearance of the tissue loss

On this supplementary test set, the CNNs achieved an overall accuracy of 0.89, a specificity of 0.96, and a sensitivity of 0.86 (Table 1). In addition, the agreement between the CNNs and each individual observer was 0.79 (*Cohen*’s kappa coefficient). In 20 cases, labels assigned by the CNNs differed from those unanimously given by all the observers. In detail, CNNs misclassified healthy ears as pathological (n = 5), pathological ears as healthy (n = 2) or affected by artefacts (n = 6), and artefacts as healthy (n = 5) or pathological (n = 2) ears. Representative examples of errors made by the CNNs are shown in Fig. 5.

## Discussion

Biting lesions are major concerns in pigs, even more after the EU ban to routine tail docking, which could exacerbate damaging behaviors in pigs [24]. In this context, a potential link between tail and ear biting has been also hypothesized, with ear biting behavior possibly occurring more frequently when tails are docked short [25].

In recent years, both the scientific community and the livestock industry have shown interest in the implementation of computer vision (mostly AI-based) at slaughtering. Such an approach could allow the systematic, standardized, and fatigue-proof detection of lesions, thus contributing to assess animal health and/or welfare on farm. As reviewed by Sandberg et al. [21], a certain attention has been focused on the pig sectors, and this is not surprising as it is among the most intensive and technological ones.

This study fits into the above research landscape. More in detail, it aimed to develop CNNs able to detect ear biting lesions, which are regarded as abattoir-based indicators of on-farm welfare. Notably, Blömke et al. [4] developed a camera-based system to assess ear and tail lesions. Their algorithm considered ear shape, surface and position, ruling out some additional findings (e.g., otohematomas, artefacts after dehairing). A consistent reliability was seen among observers, while it was lower when compared with the camera-based system [4]. This study shows some similarities with that published by Blömke et al. [4]. For instance, the aim and the main features of images are almost identical. However, it is useful to highlight a few peculiarities, which could hopefully contribute to streamline the development of more effective and efficient systems. First, we strictly focused on the evaluation of ears’ contours, aiming to enhance consistency among observers and to speed up the CNN training. Indeed, subjective and non-standardizable assessments can cause severe biases, which are difficult to be mitigated and could impair the CNNs’ performances [26]. Our investigative approach suitably solved that task, as the agreement among the observers was almost perfect and further increasing over time [27]. In addition, free open-source software and AI-powered tools were used herein with promising results. In our opinion, this is worthy of mention, as it suggests that AI-based tools could be widely adopted to address specific tasks.

The CNN training needs thousands of images, taken under various field conditions. This is crucial to manage biases and to suitably develop CNNs, which could be successfully employed in different slaughterhouses. On the other hand, the setup of cameras in several abattoirs is logistically challenging and expensive at this stage. Therefore, as already tested, we built the dataset using smartphones cameras, which are always available, easy to use, and provide suitable pictures (their resolution is usually reduced to lighten the CNN workload) [20, 22, 23]. Of course, the CNN needs to integrate with other components to fully automate the entire process, from images’ collection to sharing results with the stakeholders. In this respect, valuable experiences are being gathered, which could serve as guidelines for routinely application of AI-powered tools [21, 28].

In this study, we used the same CNN twice, assigning a single task at a time, as this approach can lead to better performance [29]. It is impossible to define universally accepted cut-off values to assess CNN capabilities, as they closely depend on the context, application, and data complexity. The CNNs developed herein achieved high values of accuracy (> 0.88), specificity (> 0.89), and sensitivity (> 0.86). Such performance is consistent with those reported in similar studies, even when compared with investigators who did not contribute to the training process. For instance, existing CNN models report sensitivity values ranging from 0.77 to 0.96, and specificity values ranging from 0.86 to 0.99 [21]. However, there is always room for improvement. This is a distinct feature of AI-based tools, which can progressively enhance their experience and skills. As a rule, CNNs’ performance can be further improved by increasing the dataset size and enhancing the balance of the dataset among the different classes (i.e., healthy *vs* lesion *vs* artefact). It is also worth noting that the CNNs were trained on images of heavy white pigs. Although ear profile is unlikely affected by its size or skin pigmentation, augmenting the dataset with pictures of lightweight and/or pigmented pigs could be beneficial to support the broader application of these CNNs.

Slaughtering procedures often induce prominent artefacts, which might complicate the detection of lesions [20, 22, 23]. As far as skin lesions are regarded, conflicting data have been reported about the effect of routine slaughtering process [30]. In our experience, artefacts represent a challenge to detect ear lesions in slaughtered pigs, both for veterinarians and for CNN. This challenge greatly varies among different abattoirs and could be solved by taking pictures before brushing, whenever needed and allowed by the slaughter plants.

## Conclusions

A broad consensus exists that postmortem inspection can yield valuable data regarding animal welfare and health. In this context, ear biting lesions are considered reliable indicators of animal welfare in pig farming. Artificial intelligence-based systems have been designed to detect and/or score lesions in slaughtered animals, convolutional neural networks representing a particularly important technique. We consider that this investigation contributes further insights in this field of research, suggesting that suitable performance can be achieved even with freely available tools, especially when the task is strictly defined and relatively simple. The setup of cameras at key points along the slaughter line, combined with the development of more effective tools, might enable stakeholders to assemble their own ideal puzzle, aiming to fulfil specific needs in a cost-effective manner.

## Data Availability

No datasets were generated or analysed during the current study.
